# Hawthorn (*Crataegus monogyna* Jacq.): A Review of Therapeutic Potential and Applications

**DOI:** 10.3390/molecules31020226

**Published:** 2026-01-09

**Authors:** Jagoda Kępińska-Pacelik, Wioletta Biel

**Affiliations:** Department of Monogastric Animal Sciences, Division of Animal Nutrition and Food, West Pomeranian University of Technology in Szczecin, Klemensa Janickiego 29, 71-270 Szczecin, Poland; wioletta.biel@zut.edu.pl

**Keywords:** antioxidants, bio-active compounds, hawthorn, health properties, nutraceutical, phenolic compounds

## Abstract

Hawthorn (*Crataegus monogyna* Jacq.) is a medicinal and nutritional plant widely recognized for its rich phytochemical composition and diverse health-promoting properties. The fruit, leaves, and flowers contain significant amounts of polyphenols, flavonoids, flavonols, phenolic acids and dye compounds with antioxidant properties that contribute to its strong antioxidant capacity. Numerous studies have demonstrated hawthorn’s beneficial effects on cardiovascular health, including regulation of blood pressure, lipid metabolism, and cardiac function. Additionally, hawthorn exhibits anti-inflammatory, antimicrobial, hypolipidemic, and antidiabetic properties, supporting its role in the prevention and management of chronic diseases. Its potential as a functional food ingredient and natural health supplement is increasingly recognized. However, further clinical trials and standardization of bioactive components are needed to confirm its efficacy, safety, and optimal dosage. Overall, hawthorn represents a valuable natural resource for promoting human health and well-being through diet and phytotherapy. Therefore, the aim of this study is to present—based on the scientific literature—the antioxidant properties of hawthorn and to assess the possibility of using this plant as a functional ingredient.

## 1. Introduction

In recent years, there has been growing interest in “forgotten” edible plant, which can be a valuable source of bioactive ingredients in a health-promoting diet. In an era of growing health awareness and the trend toward self-care, the importance of natural products in prevention and health support is increasingly emphasized, in accordance with the Hippocratic maxim: “Let food be thy medicine and medicine be thy food”. Eating properly selected foods can not only support daily bodily functions but also reduce the risk of free radical-related diseases, which are largely related to oxidative stress. Oxidative stress, resulting from the overproduction of reactive oxygen species (ROS), can lead to cellular damage and accelerate the aging process, as well as increase susceptibility to chronic diseases. One of the most effective ways to counteract the negative effects of ROS is a diet rich in antioxidants, which can neutralize free radicals and support the body’s endogenous antioxidant defenses. In this context, plant-based foods represent a valuable source of bioactive compounds with antioxidant properties, and knowledge of the negative impact of free radicals encourages the search for substances that support the body’s natural defense mechanisms [1].

Increasing attention is also being paid to plant byproducts, which can be a valuable source of antioxidants and other bioactive compounds, contributing to the sustainable use of natural resources [1]. Among these plants, hawthorn holds a special place—a somewhat “forgotten” species, yet with significant health-promoting potential. Hawthorn berries are rich in polyphenols, flavonoids, and other compounds with antioxidant properties, making them an attractive raw material for the development of functional food products that support the elimination of reactive oxygen species and reduce the risk of free radical-related diseases [2,3,4]. Recent studies have demonstrated significant morphological, biochemical, and molecular diversity in hawthorn, highlighting the potential for optimizing the nutritional and antioxidant parameters of the fruit [5,6]. Chemical and metabolomic analyses confirm the high antioxidant content of hawthorn berries, as well as their stability under various processing and infestation conditions [7,8,9].

In Europe, China, and North America, traditional applications of hawthorn have been well-documented across several cultural regions, encompassing both dietary and medicinal forms. In Europe, preparations made from leaves and flowers—often as herbal teas, tinctures, or infusions—have been used traditionally to support cardiovascular and digestive health, as well as mild nervous tension (e.g., palpitations and anxiety) in folk practice, with fruits also being processed into jams, syrups, or wine for consumption [10,11,12]. In Traditional Chinese Medicine, hawthorn fruit has been used for over two millennia both as a food ingredient and as a medicinal agent to aid digestion, improve blood circulation, and address “blood stasis”; the fruit is consumed in diverse forms such as teas, snacks, and functional beverages, and is also integrated in herbal formulations with other botanicals [13]. Indigenous and settler communities in North America have historically consumed hawthorn fruits fresh and dried, incorporated them into dietary practices and folk remedies, and used decoctions or extracts of various plant parts as traditional treatments for cardiovascular and gastrointestinal complaints [14]. These varied applications underscore not only the broad ethnobotanical relevance of hawthorn across regions but also the importance of the plant part used—fruits more often as foods and beverages, and leaves and flowers predominantly for traditional medicinal preparations.

The genus *Crataegus* L. belongs to the Rosaceae family, subfamily Maloideae. Its taxonomy is exceptionally complex—the number of species is estimated at approximately 150 to as many as 1200, depending on the taxonomic approach, due to strong hybridization [15]. Wild hawthorn species are widespread in the temperate climate of the Northern Hemisphere, including Europe and Asia. Over 300 chemical compounds have been detected in *Crataegus*, especially *C. monogyna*, including flavonoids, procyanidins, organic acids, and many other biologically active substances [16,17]. Extracts from leaves, flowers, and fruits exhibit strong antioxidant, antibacterial, cardioprotective, anti-inflammatory, and hepatoprotective properties. In vitro and in vivo studies confirm anti-inflammatory effects, liver protection, and anticancer potential [17,18,19]. *C. monogyna* leaf extract exhibits high polyphenol content and significant antioxidant activity as well as antimicrobial activity, including against *Staphylococcus aureus* [16].

The aim of this study is to present—based on the scientific literature—the antioxidant properties of hawthorn and to assess the possibility of using this plant as e.g., a functional ingredient.

## 2. Botanical Characteristics

The genus *Crataegus* L. includes many species, of which the edible ones include mainly:

- *Crataegus monogyna* Jacq.
- *Crataegus laevigata* (Poir.) DC.
- *Crataegus douglasii* Lindl.
- *Crataegus submollis* Sarg.
- *Crataegus succulenta* Schrad. ex Link
- *Crataegus azarolus* L.
- *Crataegus punctata* Jacq.
- *Crataegus sanguinea* Pall.

**Figure 1:**
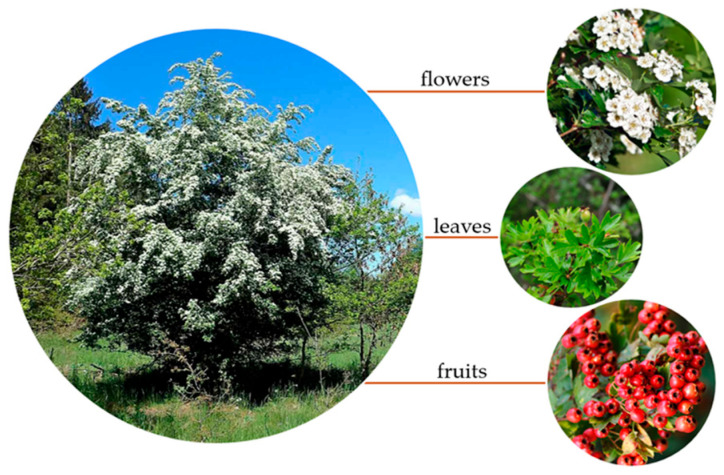
*Crataegus monogyna* Jacq. and its morphological parts.

Most of the research in the available current scientific literature focuses on *Crataegus monogyna*, which has a shrubby or low-woody habit, typically reaching a height of 5–15 m. Young shoots have smooth, light-gray bark; with age, fine cracks and furrows appear on the bark, or the bark thickens. Thorny branches—one of the characteristic features—are sharp thorns growing directly from the shoots or from short shoots, usually 1–3 cm long (although descriptions give a range of up to 6 cm). The leaves are single, helically arranged, and highly variable in morphology—often lobed (3–7 lobes), with shapes ranging from broadly ovate to rhomboid; the margins are usually serrated or entire. Both sides of the leaf exhibit differences: the upper side is light and shiny, while the lower side is lighter and slightly felt-like [20] (Figure 1). The flowers are hermaphrodite, composed of 5 sepals, 5 petals (usually white, rarely pink), a dozen or so stamens (5–25), and usually one or two pistils. They are grouped in corymbs typical of *Crataegus*, often called subumbels. Flowering occurs in late spring—May–June—and varies depending on the species. The fruit is a spherical or slightly flattened berry, ranging in color from red to purple, containing 1–5 hardened seeds (pyrene), one typical of *C. monogyna*, more in other species or hybrid forms. The berry has a persistent calyx at the top of the fruit and is often eaten by birds, which promotes their dispersal (zoochorous dispersal). Plants of the genus *Crataegus* prefer sunny or partially shaded locations, well-draining soil, and are resistant to drought and low temperatures. They often occur on forest edges, in thickets, and in ruderal areas. The flowers are pollinated by insects—primarily bees, flies, and others. Reproduction involves both cross-pollination and apomixis, which favors the development of numerous intermediate forms and hybrids [11].

## 3. Chemical Composition and Nutritional Value

Studies conducted on hawthorn from Romania have shown that the fruit contains an average moisture content of approximately 69% on a fresh weight basis, indicating its high water content and low energy density. The fruit contains 3.5% protein, placing it among the low-protein, yet still useful, raw materials due to its nutrient content. Crude fat accounts for 0.8%, indicating the presence of beneficial, albeit small, amounts of lipids. The ash content is 1.75%, confirming the presence of minerals. Hawthorn fruits are the source of both macroelements and microelements. The fruit is also characterized by an acidic pH (pH 4), which is important for its processing properties and shelf life. What is more, hawthorn has strong antioxidant properties [21,22,23,24]. Another study demonstrated that ethyl-acetone extracts of hawthorn leaves and fruit contain significantly higher concentrations of polyphenols than alcohol or chloroform extracts. Both leaves and fruit are an exceptionally rich source of antioxidants [25] (Table 1).

Studies indicate the presence of fatty acids in hawthorn fruit, particularly oleic and linoleic acids [22]. In the analysis conducted by Bernatoniene et al. [37], extraction with various solvents was used, followed by evaluation of the antioxidant activity of *Crataegus monogyna* extracts using the DPPH and ABTS methods. A clear advantage of alcoholic extracts over aqueous extracts was observed—DPPH activity increased 2.3-fold, and ABTS activity increased 2.5-fold. Chromatographic analyses showed that epicatechin and catechin were the key compounds responsible for antioxidant activity, while procyanidin B2 showed only a marginal effect [37]. In another study, Akcan et al. [38] examined hawthorn fruit extracts for their degree of inhibition of lipid oxidation in meat products. Acetone extracts rich in polyphenols (12.81 mg GAE/g fruit) significantly inhibited fat oxidation and delayed the development of off-flavors in cooked pork rings. A study by Abuashwashi et al. [39] compared the polyphenol content of aerial parts of *C. monogyna* from different regions of Spain. The total polyphenol content in methanol extracts ranged between 117 and 204 mg GAE/g extract, and antioxidant activity—measured by the DPPH method—ranged from 0.82 to 3.76 μg/mL. In a subsequent paper, Belabdelli et al. [16] showed that ethanol extracts from *C. monogyna* leaves contain as much as 473400 μg GAE/g and 80900 μg CE/g flavonoids, with IC_50_ DPPH at the level of 22.5 µg/mL, which clearly confirms their strong antioxidant and antibacterial properties (including against *Staphylococcus aureus*). Martín-García et al. [40] designed optimal conditions for the ultrasonic extraction of phenols from *C. monogyna* leaves, which allowed the identification of 19 different phenols and 11 proanthocyanidins using HPLC-MS. The most abundant derivatives were protocatechuic acid, dihydroxybenzoyl pentosides, chlorogenic acid, hyperside, isoquercetin, and monomer and dimer proanthocyanidins.

Research by Oancea et al. [41] on the lipid composition of hawthorn indicated the presence of significant amounts of polyunsaturated fatty acids (PUFAs), primarily n-3 and n-6, constituting over 57% of total fatty acids. The fruit also contains carotenoids (xanthophylls) and vitamin E, making hawthorn a valuable antioxidant source. Recent studies have documented high pigment and polyphenol content in *C. monogyna* fruit powder, as well as the presence of epicatechin, methyl ferulic acid, catechin, procyanidins B1 and B2, and carotenoids such as lutein and lycopene. These pigments demonstrated color and antioxidant stability during various food processing conditions [27].

In case of polyphenols, HPLC analysis showed epicatechin to be the main compound (~37% of total phenols), followed by vanillic acid (~23%) and quercetin (~20%), as well as trace amounts of catechin and ferulic acid, at 156 μg/g and 28 μg/g, respectively [42]. Studies on the effect of altitude confirmed the stability of essential components such as protein, fat, and fiber regardless of cultivation altitude. However, the content of micronutrients, such as calcium, was higher in samples from mountainous areas, indicating the adaptive nature of mineral accumulation [43]. A number of flavonoids, such as rutoside, apigenin, myricetin, naringenin, and kaempferol, were detected in the leaves and fruits, as well as strong antioxidant activity—the IC_50_ DPPH value was only 22.5 µg/mL, indicating the high biological activity of the leaf extracts [24]. Studies also confirm the presence of essential elements—calcium, magnesium, and iron—in amounts exceeding 1 ppm, which constitutes the basis of their nutritional and pharmacological value [22,42].

## 4. Selected Biologically Active Compounds and Their Properties

Hawthorn is characterized by a diverse array of secondary metabolites that can be broadly categorized into several major chemical classes, each with distinct structural features and biological properties. Polyphenols, including flavonoids and procyanidins, represent one of the most abundant and biologically significant groups in hawthorn. These compounds possess one or more aromatic rings with hydroxyl substituents and often occur as glycosides or oligomers. Flavonoids such as rutoside and quercetin are among the most frequently identified flavonol glycosides in hawthorn fruits and leaves [10]. Phenolic acids such as cinnamic, ferulic, gallic, and p-hydroxybenzoic acids are another important group in hawthorn phytochemistry. These compounds typically consist of a phenolic ring with a carboxylic acid group and may derive from hydroxybenzoic or hydroxycinnamic acid backbones. Phenolic acids have been detected in both fruits and leaves, where they contribute to antioxidant capacity and may influence other biological activities associated with hawthorn extracts [44]. Beyond polyphenols and phenolic acids, carotenoids represent a class of tetraterpene pigments that include compounds such as lutein and lycopene. These pigments are responsible not only for coloration in plant tissues but also possess antioxidant properties linked to health benefits. In hawthorn berries, carotenoid profiles may include lutein, α- and β-carotenes, and lycopene alongside other pigments, which together contribute to both the visual characteristics and functional bioactivity of the fruit [27].

### 4.1. Polyphenols

**Figure 2:**
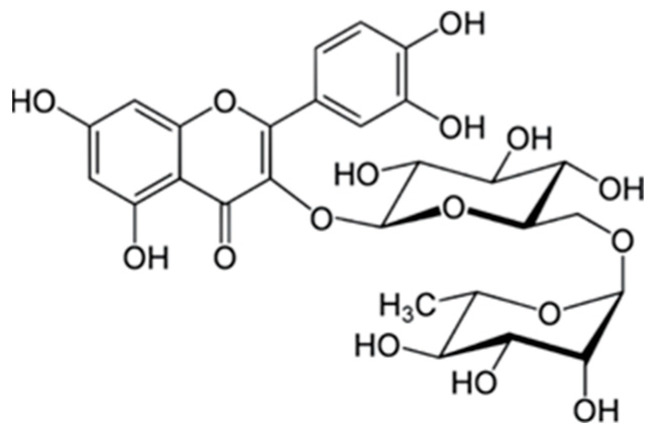
The chemical structure of rutoside [56].

Rutoside, also known as quercetin-3-O-rutinoside, a flavonol glycoside present in hawthorn, is one of the most potent antioxidant compounds identified in the plant. Its molecular structure contains multiple hydroxyl (–OH) groups—ten in total—which significantly enhance its ability to scavenge free radicals and chelate metal ions (Figure 2). The number and position of hydroxyl groups are directly associated with antioxidant potential; therefore, rutoside demonstrates stronger radical-neutralizing capacity than simpler phenolic acids such as gallic, ferulic, or cinnamic acids [45,46]. Through its powerful redox properties, rutoside protects biomolecules from oxidative damage, stabilizes cell membranes, and supports the regeneration of other antioxidants like vitamin C. Numerous studies have also highlighted its anti-inflammatory, vasoprotective, and cardioprotective effects, which contribute to hawthorn’s traditional use in cardiovascular therapy [47,48,49]. Furthermore, rutoside modulates oxidative signaling pathways, reduces lipid peroxidation, and improves capillary strength, making it a key compound responsible for the broad pharmacological benefits of hawthorn preparations. Due to its low bioavailability, rutoside is deglycosylated in the body by gut microbiota to quercetin and phenylacetic acid derivatives [50,51]. In vitro experiments indicate a strong antioxidant effect of rutoside and its glycosides, with inhibition of the production of reactive oxygen species and cytokines. The rutoside-copper complex inhibited the generation of reactive oxygen species and lipid peroxidation more effectively than rutoside alone. In in vitro studies, rutoside reduced collagen-induced platelet aggregation [51,52]. In the in vivo studies in rats and mice, rutoside significantly reduced platelet aggregation and protected against acute thromboembolism induced by collagen and epinephrine [53]. Although direct data are limited, studies suggest that rutoside has analgesic effects and may potentially act synergistically with nonsteroidal anti-inflammatory drugs and paracetamol. However, further studies are required to confirm these observations in vivo. Rutoside has been shown to have cardioprotective effects: in hypertensive rats, oral administration increased baroreceptor sensitivity, improved vascular function, and reduced oxidative stress. Improvements in lipid profile and glycemia were observed in models of diabetes, although specific doses remain to be determined [54]. In models of ischemic stroke, rutoside supported the recovery of motor function and protected neurons from degeneration through antioxidant mechanisms [55].

**Figure 3:**
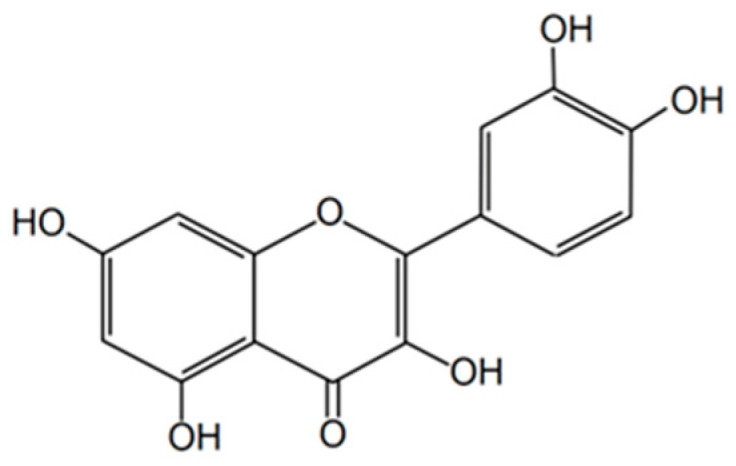
The chemical structure of quercetin [56].

Quercetin (3,3′,4′,5,7-pentahydroxyflavone), a common flavonol in hawthorn, is an important contributor to the plant’s antioxidant and cardio-protective profile; it is found especially in leaves and flowers and often occurs alongside glycosylated derivatives such as rutoside (quercetin-3-rutinoside) [24,57]. Chemically, quercetin is a pentahydroxyflavone (five phenolic −OH groups at the 3, 3′, 4′, 5 and 7 positions), and this arrangement of hydroxyls underpins its strong free-radical scavenging and metal-chelating abilities [58] (Figure 3). In general, structure–activity studies show that antioxidant potency of phenolics correlates with both the number and position of hydroxyl groups: more appropriately placed free phenolic −OH groups increase hydrogen-donating ability and radical stabilization [58]. Although glycosylated forms such as rutoside carry additional hydroxyls in their sugar moieties (so their total count of –OH groups can be numerically higher), glycosylation at key positions (for example the 3-OH) often reduces the capacity of the flavonoid aglycone to donate hydrogen and stabilize radicals; consequently, quercetin aglycone frequently displays equal or greater radical-scavenging activity than its glycosides despite having fewer total hydroxyls [57,58]. Thus, when comparing antioxidant “strength” between hawthorn samples (e.g., leaves vs. flowers), it is important to consider both the abundance of quercetin and related glycosides and the chemical form (aglycone vs. glycoside), because total hydroxyl count alone does not fully predict in-vitro or biological antioxidant effectiveness [10,24]. The bioavailability of quercetin in mammals is very low (<1%) and largely depends on the glycoside form, the dietary matrix used, and the individual gut microbiota [59,60]. In the in vitro studies with macrophages, quercetin inhibited singlet oxygen production, inducible nitric oxide synthase (iNOS) expression, cyclooxygenase-2 (COX-2), tumor necrosis factor alpha (TNF-α), and interleukin 6 (IL-6) expression via modulation of the mitogen-activated protein kinase (MAPK) and nuclear factor kappa B (NF-κB) pathways [61]. Furthermore, in a study of healthy volunteers, quercetin supplementation for 4 weeks increased total serum antioxidant levels, although it did not affect exogenous cytokines in ex vivo samples—suggesting limited anti-inflammatory effects in individuals without inflammation [62]. In a rat model with streptozotocin-induced diabetes, quercetin administered at doses up to 50 mg/kg significantly improved the enzymatic antioxidant profile, reduced lipid peroxidation, and had anti-inflammatory effects in liver and kidney tissues [63]. Quercetin was a supportive intervention in chronic cancer cachexia, reducing weight loss and IL-6 levels and improving lipid metabolism [64]. In a transgenic breast cancer model, a diet containing 0.2% quercetin significantly reduced the number and volume of tumors (−20% and −78% compared to the control group), while also specifically altering gene expression related to cellular proliferation and metabolism [65]. In a gastric cancer mouse model, oral administration of quercetin at a dose of 30 mg/kg/day for two weeks inhibited tumor growth from day 5 of observation, with significantly smaller tumor size compared to the control [66]. Liposomal quercetin administered (50 mg/kg every 3 days) for 21 days inhibited tumor growth in three cancer models (CT26, H22, LL/2) and prolonged mouse life [67].

### 4.2. Phenolic Acids

**Figure 4:**
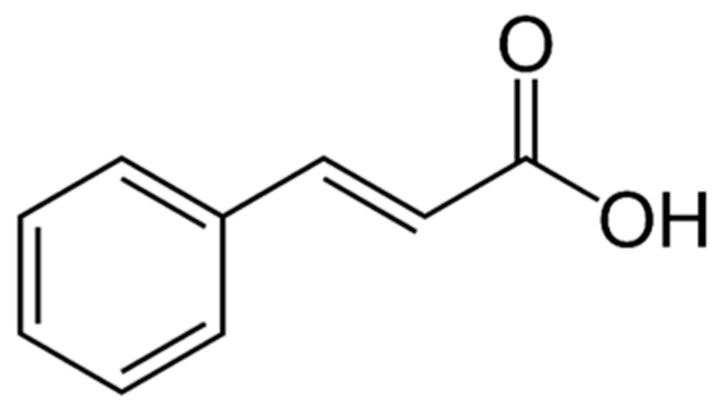
The chemical structure of cinnamic acid [56].

Organic acids are naturally occurring or synthetically produced organic compounds that have specific physiological functions. These substances contribute significantly to the taste of plants and also offer a range of health-promoting properties. They are known to have antioxidant, anti-inflammatory, and antimicrobial effects, as well as the ability to influence gut microbiota and metabolic processes [68]. Phenolic acids, such as gallic and ferulic acid, are particularly recognized for their strong antioxidant potential [69]. The levels of organic acids in plants are linked to processes like respiration, transpiration, and various metabolic activities, and they can fluctuate depending on the plant’s developmental stage [70]. Cinnamic acid (trans-cinnamic acid) (Figure 4), is a natural phenolic acid belonging to the phenylpropanoid group, widely distributed in plants. It has a characteristic honey-like aroma. Cinnamic acid and its derivatives—members of the hydroxycinnamic acid family—have been identified in extracts of hawthorn and are increasingly recognized for their contribution to the plant’s bioactive profile. HPLC-based phytochemical investigations have revealed that hawthorn extracts contain a spectrum of cinnamic acid derivatives, such as coumaroyl- and caffeoylquinic acids, which fluctuate in concentration during fruit development and contribute significantly to antioxidant capacity [71,72]. These compounds exert free-radical scavenging and metal-chelating actions, supporting hawthorn’s traditional uses in cardiovascular health and oxidative-stress-related conditions. For example, the presence of cinnamic acid derivatives aids the extract’s ability to reduce lipid peroxidation and protect vascular endothelium under stress [47]. Collectively, the identification and quantification of cinnamic acid-type phenolics in hawthorn reinforce its status as a multifaceted phytopharmaceutical resource, particularly in formulations aimed at antioxidant and vascular support. Significant results come from the study by Liu et al. [73], which demonstrated that cinnamic acid can induce cytostasis and reverse the malignant characteristics of human cancer cells (glioma, melanoma, prostate cancer, and lung cancer) in vitro. Morphological signs of differentiation and increased melanin production, decreased invasive capacity (including reduction of type IV collagenase and the TIMP-2 regulator), as well as altered expression of class I antigens, were observed in melanoma cells. The mechanism of cinnamic acid’s anticancer action involves blocking protein isoprenylation, which inhibits mitogenic signaling [73]. However, another cell line (e.g., HT-144 melanocytes) exposed to cinnamic acid showed an increase in the frequency of nuclear aberrations, indicating a potential genotoxic effect at higher concentrations [74]. A study in rats fed a high-fat diet and administered cinnamic acid doses (30 mg/kg/day for 7 weeks) demonstrated numerous therapeutic effects: reduced body weight gain, lower total cholesterol and triglyceride levels, reduced pancreatic lipase activity, and restoration of aortic and arterial diameters to near-control values. Loss of hepatic steatosis and improvement in liver and kidney toxicity markers were considered clinically significant cardioprotective effects of cinnamic acid [75]. A literature review confirmed that cinnamic acid has strong antioxidant, anti-inflammatory, antihyperglycemic, antiatherosclerotic and vasodilatory properties, which are valuable in the context of cardiovascular diseases and metabolic disorders [76].

**Figure 5:**
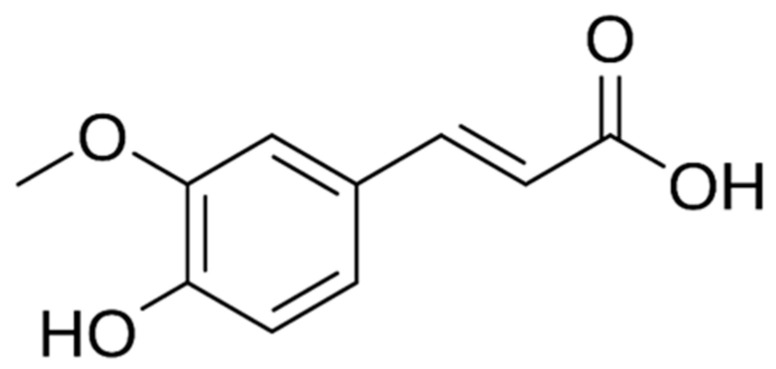
The chemical structure of ferulic acid [56].

Ferulic acid (FA, 4-hydroxy-3-methoxycinnamic acid) (Figure 5), is a phenolic acid from the hydroxycinnamic acid group, widely found in the cell walls of many plants, where it is often covalently bound to hemicellulose, which influences its bioavailability. It is another key phenolic compound identified in the fruits, leaves, and flowers of hawthorn, contributing to the plant’s antioxidant and therapeutic properties. As a hydroxycinnamic acid derivative, ferulic acid plays a significant role in neutralizing free radicals and protecting cellular components from oxidative damage [77]. It has been shown to enhance the overall antioxidant potential of hawthorn extracts and to act synergistically with other polyphenols, such as gallic and chlorogenic acids, in mitigating oxidative stress [24]. Beyond its antioxidant capacity, ferulic acid exhibits anti-inflammatory, antimicrobial, and cardioprotective effects, supporting vascular health and reducing lipid peroxidation [78]. Recent studies also highlight its potential neuroprotective and anticancer properties through modulation of signaling pathways associated with inflammation and apoptosis [79]. Studies have shown that ferulic acid effectively scavenges hydroxyl radicals, reactive singlet oxygen, and superoxide radicals, surpassing eugenol in many assays. Ferulic acid’s cytotoxicity was approximately 10-fold less toxic than eugenol [80]. In an in vitro genotoxicity model, FA at concentrations ranging from 50 to 1500 µM did not induce DNA damage [81]. In vitro and in vivo studies, ferulic acid demonstrated antithrombotic effects: it inhibited thrombin- or collagen/epinephrine-stimulated platelet aggregation, reduced clot retraction, and delayed clotting times (intrinsic and extrinsic pathways). In vivo, it significantly reduced thrombosis in a model of acute thromboembolism [82]. In a rat diabetes model, ferulic acid administration at doses of 150 and 300 mg/kg restored antioxidant levels. It also corrected the lipid profile (cholesterol and triglycerides) better than metformin, demonstrating potential in the treatment of diabetic cardiomyopathy [83]. A review of studies in animal models of Alzheimer’s disease showed that ferulic acid significantly improves spatial memory and reduces amyloid-β (Aβ_1–40_, Aβ_1–42_) plaque deposition. Mechanisms include antioxidant, anti-inflammatory, mitochondrial protection, and inhibition of apoptosis [84]. In a rat model of ischemic stroke, ferulic acid (100 mg/kg) was shown to prevent the decline of metabolic enzymes and inhibit the increase of pyridoxal phosphatase, suggesting mechanistic protection of neurons after ischemic injury [85].

**Figure 6:**
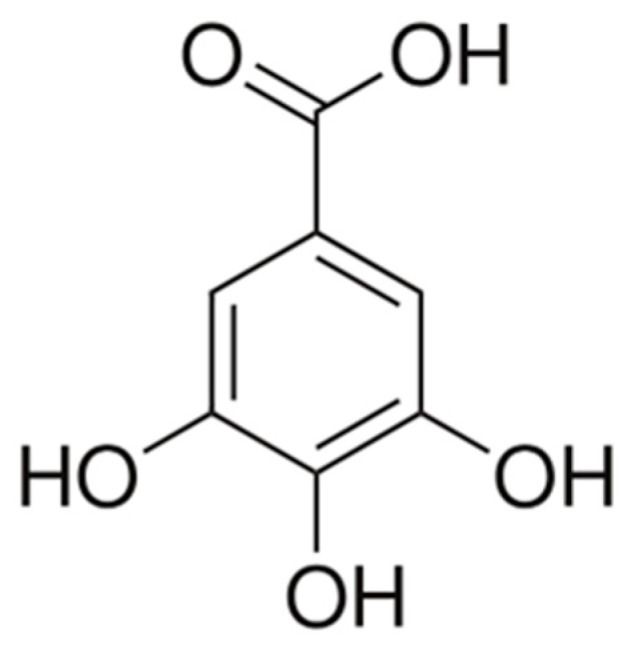
The chemical structure of gallic acid [56].

Gallic acid (3,4,5-trihydroxybenzoic acid) (Figure 6), a phenolic organic acid commonly found in various parts of the hawthorn, has been extensively studied for its wide range of biological activities and contribution to the plant’s medicinal properties. Research indicates that hawthorn fruits, leaves, and flowers contain measurable levels of gallic acid, which significantly contribute to the plant’s strong antioxidant capacity [24,79]. As a potent free radical scavenger, gallic acid helps protect cells against oxidative stress, a key factor in aging and the development of chronic diseases. Beyond its antioxidant role, gallic acid exhibits anti-inflammatory, antimicrobial, and cardioprotective effects. It has been shown to enhance endothelial function, reduce lipid peroxidation, and support cardiovascular health [78]. Additionally, emerging evidence suggests that gallic acid possesses anticancer potential through the modulation of signaling pathways that regulate cell proliferation and apoptosis [77]. Overall, gallic acid extracted from hawthorn represents a bioactive compound of considerable therapeutic importance, reinforcing the traditional use of hawthorn in herbal medicine for promoting heart and general health. In studies on human cancer cell lines, gallic acid reduced cell survival in a dose-dependent manner and induced apoptosis. In mice, administration of a gallic acid solution in water (0.3–1% *w*/*v*) for 6 weeks significantly inhibited tumor growth, reduced the number of proliferating cells, increased the number of apoptotic cells, and limited microangiogenesis [86]. In a mouse lung cancer model (LL-2 line), administration of gallic acid in water (1 mg/mL) in combination with cisplatin significantly reduced tumor mass after 29 days compared to control and single therapy, while simultaneously increasing apoptosis [87]. In colorectal cancer models, gallic acid (200 mg/kg every other day for 38 days) inhibited tumor progression by reducing Ki-67 activity, and the expression of genes such as c-MYC was reduced, suggesting a mechanism of inhibition of proliferation through DNA binding [88,89]. In MCF-7 cells (breast cancer), gallic acid at concentrations of 2.5–10 µg/mL synergistically increased cytotoxicity in combination with cisplatin, while showing no toxicity to healthy cells, suggesting a reduction in the genotoxic effect of chemotherapy [90]. Data from an oral squamous carcinoma model have shown that gallic acid inhibits cancer cell proliferation, migration, and invasiveness, including under hypoxic conditions [88,89,90,91]. In studies on mouse models of cyclophosphamide poisoning, gallic acid exerted a genoprotective effect: it reduced the incidence of DNA damage, protected the liver by increasing superoxide dismutase activity and glutathione levels, and ameliorated histopathological changes [90]. Gallic acid modulates numerous signaling pathways, which contributes to the inhibition of cancer cell invasiveness and proliferation, for example, in models of breast cancer and osteosarcoma [92,93]. In an oral cancer model, gallic acid increased E-cadherin expression, which limited cell migration and tumor growth [89].

**Figure 7:**
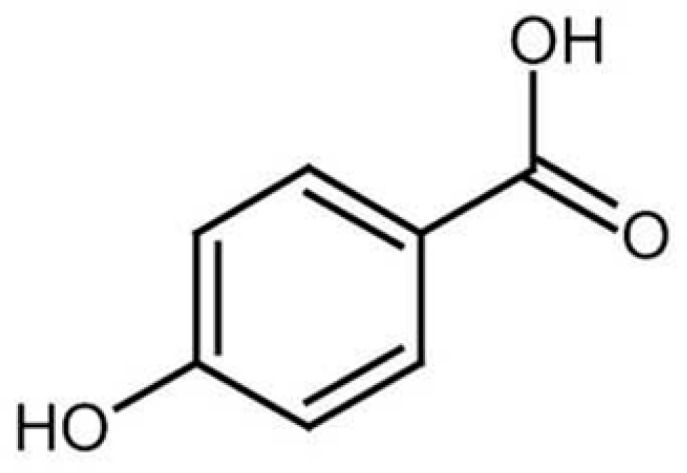
The chemical structure of p-hydroxybenzoic acid [56].

The p-hydroxybenzoic acid (PHBA) (Figure 7), also known as 4-hydroxybenzoic acid, is a model phenolic compound and a monohydroxybenzoic acid, a phenolic derivative of benzoic acid. It is isomeric with 2-hydroxybenzoic acid, known as salicylic acid, a precursor to aspirin. PHBA is a significant component in *C. monogyna* extracts, particularly in the leaf-flower portion, contributing to its antioxidant properties. Although no study has analyzed the properties of isolated p-hydroxybenzoic acid from hawthorn, the antioxidant activity of the whole extract strongly correlated with the content of phenols, including PHBA [29]. Similarly, studies on *C. monogyna* fruit extracts have confirmed that alcoholic extracts have stronger antioxidant potential than aqueous extracts—this is mainly due to the presence of epicatechin and catechin, although phenolic acids also play a role [37]. Previous studies provide strong evidence for the contribution of p-hydroxybenzoic acid to the phenolic profile of hawthorn, especially in leaf and flower extracts, and indicate its involvement in antioxidant mechanisms. However, there are no studies examining the effects of PHBA alone, which prevents an assessment of its individual health effects. Based solely on multicomponent extracts, it is difficult to clearly attribute specific effects to this acid alone.

### 4.3. Anthocyanins

Among compounds with antioxidant properties, not only phenolic compounds but also other groups of bioactive substances occupy a significant place, including coloring compounds with antioxidative properties, such as carotenoids. Although both polyphenols and carotenoids contribute to the neutralization of reactive oxygen species, their mechanism of action is different. Polyphenols, due to the presence of hydroxyl groups, donate a hydrogen atom or electron, converting free radicals into less reactive forms, which is a classic ROS scavenging mechanism [1,3]. Carotenoids, as fat-soluble tetraterpenoid pigments, act primarily by physically quenching singlet oxygen, deactivating free oxygen radicals, and stabilizing the biological membranes in which they are located [2,9].

**Figure 8:**
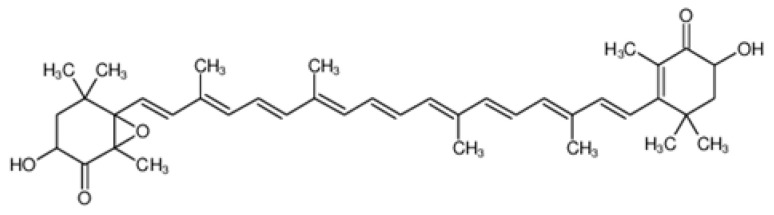
The chemical structure of lutein [56].

Lutein (C_40_H_56_O_2_) (Figure 8) is a tetraterpenoid pigment from the xanthophyll group—oxygen-containing carotenoids—that is not synthesized by mammals and must be obtained through diet. It is found in fruits, vegetables, and plant materials such as hawthorn, where its presence has been observed in several species [41]. Lutein is characterized by a blue-absorbing wavelength and the presence of hydroxyl groups on both rings, which determines its antioxidant properties and its ability to penetrate the blood-droplet barrier into the retina. Due to the relatively high lutein content in selected *Crataegus* species, the fruit of this plant may be an attractive raw material for the development of functional foods or nutritional supplements focused on antioxidant activity and tissue protection. Because lutein’s mechanism of action (lipid phase, cell membranes, reduced risk of lipid oxidation) differs from that of phenolic compounds (which predominate in the aqueous phase and act through H/electron donation), including lutein in the analysis of raw materials—such as hawthorn—allows for a more comprehensive picture of the antioxidant potential of a given material. It is also worth noting that the choice of species and cultivation conditions can significantly affect lutein content [94]. Studies on BV-2 microglial cells have confirmed that lutein effectively inhibits the production of reactive oxygen species following exposure to H_2_O_2_, increases the secretion of anti-inflammatory interleukin-10, and reduces TNF-α. Additionally, a significant reduction in proinflammatory cytokines (IL-6, TNF-α, IL-8) was found in microglia and BV-2 cell models at concentrations ≥ 4 µm [95,96,97]. In a mouse model of endotoxin-induced uveitis, lutein administration protected photoreceptors, demonstrating neuronal protection and anti-inflammatory effects in the retina [98]. In vitro, in Müller-retinal cells subjected to hypoxia, lutein blocked NF-κB and COX-2, suggesting a mechanistic anti-inflammatory and antiapoptotic effect in retinal ischemic diseases [99]. Recent data indicate that the combination of lutein and dimethylfumarate in a mouse model of remyelination enhances anti-inflammatory and neuroprotective effects compared to single preparations, suggesting synergism between antioxidant and immunomodulatory mechanisms [100]. A meta-analysis showed that lutein supplementation reduces aortic cholesterol levels and oxidized LDL concentrations, suggesting antiatherosclerotic effects [101]. Additionally, ex vivo studies on lymphocytes from patients with coronary artery disease showed that lutein reduced IL-6 secretion, confirming its anti-inflammatory effects in the context of cardiovascular inflammation [102]. Clinical studies are examining the effect of lutein on metabolic parameters in individuals with obesity [103]. In studies using hepatotoxicity models, lutein activates Nrf2 and increases the expression of antioxidant enzymes, while counteracting liver inflammation and fibrosis [104]. Lutein, being a lipophilic compound, is located primarily in the retina, brain, liver, and skin. Its absorption capacity depends on the presence of dietary fat and interactions with other phytochemicals, which can either facilitate or inhibit its absorption (e.g., anthocyanins) [105,106].

**Figure 9:**
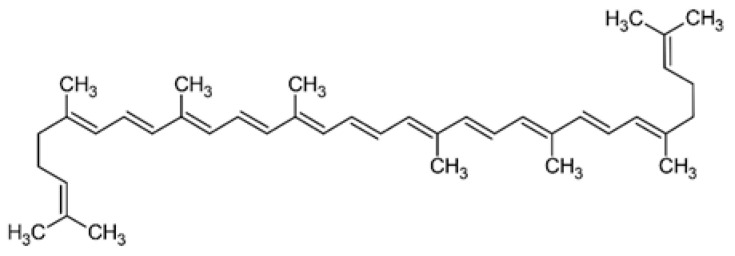
The chemical structure of lycopene [56].

Lycopene is a tetraterpene carotenoid found primarily in red and pink fruits. Its structure contains 11 conjugated double bonds, which is responsible for its strong antioxidant properties [107] (Figure 9). It is a red carotenoid from the carotene group—terpenoid compounds with strong antioxidant properties. Unlike lutein, lycopene does not contain oxygen atoms, which determines its high lipophilicity and effectiveness in neutralizing reactive oxygen species in lipid environments. Lycopene is one of the most effective natural singlet oxygen quenchers—its activity in this regard is up to ten times higher than that of vitamin E. This compound is widely studied in the context of protecting against oxidative diseases [108]. Lycopene bioavailability is enhanced by thermal processing and the presence of fats, which facilitate the breakdown of the plant matrix and isomerization from trans to cis forms, which are better absorbed [109]. Chromatographic analyses have shown that lycopene concentrations in *Crataegus* species range from 0.45 to 0.92 mg/g DW, depending on the species and fruit ripeness [94]. These studies also indicate that lycopene is a significant component of the carotenoid profile of hawthorn fruit, alongside lutein, α-cryptoxanthin, and β-cryptoxanthin. Lycopene content depends largely on environmental conditions, fruit ripeness, sunlight intensity, and drying method. Increased concentrations were observed in intensely red fruit, which correlates with its pigmentary and protective functions in plant tissues [6,94]. Lycopene is one of the most effective natural singlet oxygen quenchers [110]. In living organisms, doses of 25–50 mg/kg reduced carrageenan-induced edema and reduced markers of liver damage and lipid peroxidation, while simultaneously increasing superoxide dismutase and reduced glutathione. Similarly, in a model of cardiac ischemia, lycopene administration (1 mg/kg for 31 days) resulted in protection against oxidative stress and preservation of myocardial structure [111,112,113]. High lycopene concentrations or intake are associated with a reduced risk of stroke, cardiovascular disease, and mortality [114,115]. In a randomized clinical trial, natural lycopene supplementation in individuals with impaired endothelial function improved endothelium-dependent vasodilation by 50–60% without changes in lipids. However, in the population of healthy volunteers, the effects were insignificant, which indicates targeted efficacy in risk groups [115,116].

## 5. Medicinal Properties of Hawthorn

For centuries, hawthorn has been used in traditional medicine to support heart function, improve digestion, and strengthen the immune system. Modern medicine’s interest in this plant stems from the presence of numerous biologically active compounds, discussed in the previous sections.

### 5.1. Impact on Lipid Metabolism

A number of studies in animal models have assessed the effects of hawthorn extracts on lipids and markers of oxidative stress. Shao et al. [117] compared the effects of aqueous and ethanol extracts of hawthorn fruit in rats with induced hyperlipidemia; the ethanol extract, containing approximately 3.9 times more phenols, demonstrated a significant reduction in total cholesterol and triglycerides, an increase in antioxidant enzyme activity, and a decrease in lipid peroxidation marker levels. Hawthorn procyanidins administered to rats in a model of non-fatty liver disease improved the lipid profile, limited lipid accumulation in the liver, normalized the composition of the gut microbiota (growth of Akkermansia, Bacteroides), and reduced inflammation [118]. In a mouse model of nonalcoholic fatty liver disease, hawthorn berry polysaccharides corrected intestinal dysbiosis, strengthened the intestinal barrier, and activated the adenosine monophosphate–activated protein kinase and peroxisome proliferator-activated receptor alpha pathway, resulting in improved fatty acid metabolism [119]. Kwok et al. [120] demonstrated that hawthorn consumption has a generally beneficial effect on reversing the harmful changes associated with a high-cholesterol diet.

As demonstrated by Zhang et al. [121], hawthorn berry extract can inhibit the progression of atherosclerosis in rats fed a high-fat diet. Possible mechanisms of action include improved lipid metabolism, reduced inflammatory cytokine responses, and endothelial protection.

A combination of hawthorn leaf extract and citrus peels has been shown to be effective and safe in reducing body fat and body weight, and regulating blood lipid and leptin levels in overweight or moderately obese individuals. Moreover, they may be used to prevent the development of moderate obesity, hypertriglyceridemia and leptin resistance in overweight people [122].

### 5.2. Benefits for the Cardiovascular System

Hawthorn is a plant that has long been used in phytotherapy for cardiovascular diseases. Among the key biologically active compounds present in its raw material are flavonoids, including vitexin and its derivatives, which significantly contribute to the cardioprotective effects of hawthorn extracts. Research by Martino et al. [123] demonstrated that vitexin is one of the main flavonoids present in the leaves and flowers of *C. monogyna*, and its content in the extracts depends largely on the extraction method used. The authors emphasized that the appropriate selection of extraction conditions allows for obtaining high vitexin concentrations, which has a direct impact on the effectiveness of preparations used in circulatory diseases such as heart failure, hypertension, and coronary artery disease. More recent research by Gavrila et al. [35] confirms the importance of modern methods for obtaining phenolic compounds from hawthorn, including microwave-assisted extraction. Optimized extraction has been shown to increase the content of polyphenols, including flavonoids such as vitexin, while maintaining their biological activity. These compounds possess strong antioxidant potential, which is particularly important in the pathogenesis of cardiovascular disease, where oxidative stress plays a key role in endothelial damage and the progression of atherosclerosis. The importance of vitexin is also confirmed by studies of the phenolic composition of *Crataegus monogyna* extracts conducted by Amrati et al. [17]. The authors demonstrated that flavonoids, including vitexin, are responsible for the extract’s pronounced anti-inflammatory effects. This effect has significant cardioprotective implications, as chronic inflammation is a key factor in the development of cardiovascular disease. Limiting the inflammatory response and neutralizing free radicals helps improve the function of blood vessels and protect the heart muscle.

The anti-inflammatory and antioxidant properties of hawthorn leaf extract were assessed in isolated rat thoracic aortas. Hawthorn leaf extract significantly reduced the levels of oxidative and inflammatory markers (among others TNF-α, and IL-1β) in the aortas, confirming its potential as a protective agent for the cardiovascular system [124]. Due to its strong antioxidant properties, *C. monogyna* fruit extract has therapeutic potential for rat myoblasts [125]. A study in 110 hypertensive patients demonstrated that adding hawthorn drops to standard therapy reduced systolic blood pressure (SBP) and mean arterial pressure (MAP). Differences between groups were significant only in the sixth week of therapy, becoming less significant thereafter. The results suggest that hawthorn may have a beneficial effect on SBP and MAP, but further studies with larger numbers of participants are necessary [126].

Cathepsin cysteine proteases, such as cathepsin S, are responsible for the adverse and irreversible dysregulation of extracellular matrix proteolysis observed in cardiomyopathy, valvular heart disease, and atherosclerosis. An organic extract of *Crataegus monogyna* has been shown to play a key role in inhibiting cathepsin S in vitro and may serve as a viable source for isolating cathepsin S inhibitors [127].

Moreover, ethanol extracts of hawthorn significantly inhibited carrageenan-induced mouse tail thrombosis. Based on these results, it was concluded that these extracts may potentially be used as therapeutic agents or complementary treatments against thrombosis [128].

### 5.3. Neuroprotection

A study by Saoudi et al. [129] assessed the protective effects of an aqueous hawthorn extract in rats exposed to a combination of the pesticides deltamethrin and chlorpyrifos. The extract, rich in phenolic compounds, was administered for 10 days before pesticide exposure. The results showed that it alleviated neurobehavioral impairments, reduced DNA damage in the brain, limited lipid peroxidation, and restored the activity of antioxidant enzymes to normal levels. Hawthorn demonstrated comparable effectiveness to vitamins C and E in protecting the brain from pesticide-induced damage.

Hawthorn extracts have been shown to inhibit enzymes associated with neurodegenerative diseases, such as acetyl- and butyrylcholinesterase and tyrosinase. The results suggest that hawthorn extracts may have neuroprotective potential and merit further investigation, particularly in the treatment of Parkinson’s disease and Alzheimer’s disease [130].

Acute hypobaric hypoxia (at an altitude of 7500 m) leads to oxidative stress in the brain, damaging neurons and reducing their electrical activity. A study by Karapetyan et al. [131] assessed the effect of *Crataegus* extract as a natural antioxidant. The results showed that the extract reduced lipid peroxidation and protein amidation in the cerebral cortex and hypothalamus by reducing malondialdehyde concentrations. Thus, hawthorn may protect the brain from the negative effects of hypoxia. Furthermore, topical application of hawthorn may protect rat brain tissue from ischemia-reperfusion-induced brain damage [132].

### 5.4. Anticarcinogenic Effects

Methanol extract from hawthorn fruit exhibits cytotoxic activity against breast cancer cells regardless of hormone receptor status (MCF-7 and MDA-MB-231), without damaging normal cells. It arrests the cell cycle in the G1/S phase and inhibits the signaling pathway by regulating the expression of its agonists and antagonists. The results suggest that hawthorn may also inhibit tumor growth at the cancer stem cell level [133].

A study by Barzegarparay et al. [134] demonstrated that bioactive compounds contained in *Crataegus monogyna* (including luteolin, apigenin, and quercetin) may influence breast cancer development by interacting with the MMP9 and PPARG genes. Network pharmacology, bioinformatics, gene expression analysis, and molecular modeling methods were used, confirming hawthorn’s anticancer potential. The results suggest that *C. monogyna* has multifaceted effects and may be a promising source of natural anticancer compounds.

Żurek et al. [19] assessed the effect of hawthorn fruit, leaf, and flower extracts on the viability and invasive potential of the highly aggressive human glioblastoma multiforme cell line U87MG. Treatment with the extracts induced cytotoxic effects, the strongest for fruit extracts. All extracts not only promoted apoptosis-associated cleavage of poly(ADP-ribose) polymerase 1 but also significantly inhibited the activity of the pro-survival kinases focal adhesion kinase and protein kinase B, indicating suppression of the proliferative and invasive potential of the glioblastoma multiforme cells studied. Furthermore, hawthorn fruit extract was found to demonstrate significant activity against the A549 lung cancer cell line [34].

## 6. Conclusions

Literature on the subject confirm that *Crataegus monogyna* Jacq. extracts, especially from the fruit of this plant, have a beneficial effect on lipid profile and antioxidant activity. Hawthorn is a nutritionally rich and pharmacologically potent plant with wide-ranging health benefits, particularly in cardiovascular protection. Its diverse bioactive compounds contribute to antioxidant, anti-inflammatory, and metabolic effects that support disease prevention. Despite extensive traditional use and promising research findings, more standardized clinical studies are necessary to validate therapeutic claims and ensure consistent quality in hawthorn-based products.

## Figures and Tables

**Table 1:** Chemical composition of hawthorn (*C. monogyna*).

Component	Part of the Plant	Content	References
Main components			
Moisture	Fruit	68–69% DW	[7,21]
Crude protein	Fruit	3.06–3.50% DW	[7,21]
Crude fat	Fruit	0.80–1.22% DW	[7,21]
Crude ash	Fruit	1.75–2.77% DW	[7,21]
Total carbohydrates	Fruit	24.81–91.99 DW	[21,26]
Fatty acids			
Lauric acid	Fruit	0.24–1.91% FA	[22,26]
Palmitic acid	Fruit	10.50–13.73% FA	[22,26]
Linoleic acid	Fruit	17.53–49.6% FA	[22,26]
Oleic acid	Fruit	13.92–30.6% FA	[22,26]
Stearic acid	Fruit	2.40–2.43% FA	[22,26]
Eicosanoic acid	Fruit	1.28–1.63% FA	[22,26]
Macro- and microelements			
Ca	Fruit	58.04–126.39 mg/100 g	[7,21]
Mg	Fruit	27.83–93.49 mg/100 g	[7,21]
Na	Fruit	5.71–26.40 mg/100 g	[7,21]
Cu	Fruit	0.32–0.87 mg/100 g	[7,21]
Fe	Fruit	0.08–6.22 mg/100 g	[7,21]
Se	Fruit	0.16–0.49 mg/100 g	[7,21]
Zn	Fruit	0.09–0.59 mg/100 g	[7,21]
Polyphenols			
Total polyphenol	Fruit	9350–9570 μg GAE/g DW	[7,27]
Total polyphenol	Flowers	1155.4 μM GAE/g DW	[28]
Total polyphenol	Leaves	343540–365110 μg GAE/g DW	[25,29]
Flavonoids			
Total flavonoids	Fruit	392–6250 μg GAE/g DW	[27,30]
Total flavonoids	Flower	5398–21700 μg/g DW	[24,26]
Total flavonoids	Leaves	89780–122980 μg RUE/g DW	[24,25]
Flavonols			
Flavonols	Fruit	2113.27–4880 μg QEE/g DW	[27,31]
Rutoside	Fruit	15.70–745 μg/g DW	[5]
Rutoside	Flower	340–2115 μg/g DW	[24,32]
Rutoside	Leaves	512–1670 μg/g DW	[24,32]
Quercetin	Fruit	17.98–50.01 μg/g DW	[33,34]
Quercetin	Flower	92690 μg/g DW	[24]
Quercetin	Leaves	106240 μg/g DW	[24]
Myricetin	Fruit	27.98 μg/g DW	[33]
Myricetin	Flower	Trace	[24]
Myricetin	Leaves	47 μg/g DW	[24]
Kaempferol	Fruit	15.99 μg/g DW	[33]
Kaempferol	Flower	Trace	[24]
Kaempferol	Leaves	2 μg/g DW	[24]
Hyperoside	Leaves	115.90–136.60 μg/g DW	[35]
Flavan-3-ols (flavanols)			
Procyanidins	Fruits	26.15 μg/g DW	[30]
Catechin	Fruit	43–1210 μg/g DW	[6,27]
Epicatechin	Fruit	81–1168 μg/g DW	[6,27]
Flavones			
Apigenin	Flower	6 μg/g DW	[24]
Apigenin	Leaf	10 μg/g DW	[24]
Naringenin	Fruit	0.26 μg/g DW	[34]
Naringenin	Flower	18 μg/g DW	[24]
Naringenin	Leaves	4 μg/g DW	[24]
Vitexin	Leaves	42.50–104 μg/g DW	[35]
Phenolic acids			
Cinnamic acid	Fruit	3880 μg CAE/g DW	[27]
Ferulic acid	Fruit	11–400 μg/g DW	[5,6,27]
p-hydroxybenzoic acid	Fruit	9–360 μg/g DW	[5,27]
Vanillic acid	Fruit	5–719 μg/g DW	[6,27]
Anthocyanins			
Total anthocyanins	Fruit	21.3 μg/g DW	[36]
Total carotenoids	Fruit	21150–420 μg/g DW	[27,36]
Delphinidin	Fruit	17.23 μg/g DW	[33]
Cyanidin	Fruit	19.71 μg/g DW	[33]
Mutatoxanthin	Fruit	0.62 μg/g DW	[27]
Lutein	Fruit	0.64 μg/g DW	[27]
α-cryptoxanthin	Fruit	0.45 μg/g DW	[27]
β-cryptoxanthin	Fruit	0.46 μg/g DW	[27]
cis-β-carotene	Fruit	0.28 μg/g DW	[27]
Lycopene	Fruit	0.27 μg/g DW	[27]

DW—dry weight, FA—fatty acids, GAE—gallic acid equivalent, RUE—rutoside equivalent, QEE—quercetin equivalent.

## Data Availability

No new data were created or analyzed in this study. Data sharing is not applicable to this article.

## Abbreviations

The following abbreviations are used in this manuscript:

CE	catechin equivalent
COX-2	cyclooxygenase-2
DW	dry weight
FA	fatty acids
GAE	gallic acid equivalent
IL-6	interleukin-6
MAP	mean arterial pressure
MAPK	mitogen-activated protein kinase
NF-κB	nuclear factor kappa B
PHBA	p-hydroxybenzoic acid
PUFAs	polyunsaturated fatty acids
QEE	quercetin equivalent
ROS	reactive oxygen species
RUE	rutoside equivalent
SBP	systolic blood pressure
